# PR interval duration is associated with the presence of white matter hyperintensities: Insights from the epidemiologic LIFE-Adult Study

**DOI:** 10.1371/journal.pone.0269815

**Published:** 2022-06-15

**Authors:** Jelena Kornej, Katrin Friedrich, Matthias L. Schroeter, A. Veronica Witte, Maryna Polyakova, Arno Villringer, Markus Löffler, Samira Zeynalova

**Affiliations:** 1 School of Medicine – Cardiovascular Medicine, Boston University, Boston, Massachusetts, United States of America; 2 LIFE – Leipzig Research Center of Civilization Diseases, University of Leipzig, Leipzig, Germany; 3 Institute for Medical Informatics, Statistics, and Epidemiology, University of Leipzig, Leipzig, Germany; 4 Max Planck Institute for Human Cognitive and Brain Sciences & Clinic for Cognitive Neurology, Leipzig, Germany; Universitatsklinikum Wurzburg, GERMANY

## Abstract

**Background:**

PR interval prolongation is a preliminary stage of atrial cardiomyopathy which is considered as an intermediate phenotype for atrial fibrillation (AF). AF is a known risk factor for cerebrovascular adverse outcomes including stroke. Cerebral ischemia is one cause of white matter hyperintensities (WMHs), and cognitive dysfunction.

**Aim:**

To analyze the relationship between PR interval and WMHs.

**Materials and methods:**

We performed a cross-sectional analysis with individuals from the LIFE-Adult-Study (a population-based cohort study of randomly selected individuals from Leipzig, Germany) with available brain MRI and ECG. The Fazekas stages were used to quantify WMHs (0 = none; 1 = punctate foci; 2 = beginning confluence; 3 = large confluent areas). Stages 2–3 were defined as advanced WMHs. The PR interval was measured from resting 12-lead ECG. PR duration >200ms was defined as PR interval prolongation. We used a binary logistic regression for statistical analysis. We examined the relationship between MRI and ECG measures and adjusted them for clinical risk factors.

**Results:**

We included 2464 individuals (age 59±15 years, 47% women) into analyses. The median PR interval was 160ms (interquartile range 143–179), and 319 (13%) individuals with advanced WMHs, were significantly older, had more cardiovascular comorbidities and risk factors compared to individuals without WMHs (all p<0.005). On univariable analysis, PR interval duration (OR 1.01, 95%CI 1.01–1.02, p≤0.001) and PR interval ≥160 ms (OR 2.1, 95%CI 1.6–2.7, p≤0.001) were associated with advanced WMHs. In multivariable analysis, while PR interval duration was not associated with WMHs in the whole cohort, individuals with PR ≥160ms had higher risk for WMHs.

**Conclusion:**

PR interval duration is associated with advanced WMHs beside advanced age, hypertension, and history of stroke. Further research is needed to determine whether changes in PR interval indices are clinically relevant for changes in WMHs.

## Introduction

The life expectancy of the populations in industrialized countries has continued to increase. [1]. Maintaining cognitive abilities is of immense importance for healthy aging and active participation in social life. White matter Hyperintensities (WMHs) represent structural and functional changes in the brain and are often found in older adults [2]. WMHs are significantly associated with impairments in cognitive function and range from 27% to 87% in populations older than 65 years in cross-sectional studies, conducted in four US communities by the Cardiovascular Health Study [3]. Increased WMHs, brain atrophy, and volume loss are clinically linked to several markers of cognitive decline in community-based populations [4–7].

Impaired cognitive function is often seen in patients with cardiovascular diseases, including chronic heart failure (HF) [8]. Recent studies reported an association between HF and structural brain changes, including WMHs [9, 10]. Atrial fibrillation (AF) is also associated with accelerated cognitive decline and higher risk of dementia [4, 11, 12]. The pathomechanisms have not been completely elucidated, but may involve subclinical cerebral infarctions or micro-infarctions, chronic cerebral hypoperfusion, inflammation, and shared vascular risk factors, such as arterial hypertension and diabetes mellitus [13]. Hypertension is the most important risk factor for cardiovascular diseases. In Germany every third citizen has a diagnosed hypertension [14]. Hypertension increases the risk of atrial fibrillation with age and enlargement of the mass and size of the left atrium and predisposes to its chronification [15]. Permanently increased pressure conditions in the heart during hypertension lead to atrial cardiomyopathy, which increases the risk of conduction disorders and arrhythmias such as AF [16].

The electrocardiographic P-wave and PR interval mirror atrial and atrioventricular conduction. Recent studies demonstrated an association between PR interval prolongation and underlying atrial remodelling leading to AF [17, 18], while P-wave duration was associated with cardiovascular and all-cause mortality [19]. Also, PR interval prolongation was associated with electro-anatomical substrate in AF patients and rhythm outcomes after catheter ablation, assuming that PR interval could be used as a marker for atrial remodelling [20, 21]. Previously we demonstrated that individuals with PR interval prolongation and AF shared similarities in echocardiographic parameters, renal function, and blood biomarker levels, confirming the assumption that PR interval prolongation might be considered as a preliminary (intermediate) stage for AF [22]. However, the association between PR interval prolongation and structural brain changes (WMHs) is understudied. Therefore, the aim of the current study was to analyze the association between PR interval duration and advanced WMHs. We hypothesized that PR interval prolongation was associated with advanced WMHs stages.

## Methods

### Study population

The study comprised an age and gender stratified random sample of residents, age 20–79 years, in Leipzig, Germany [23]. As part of the Leipzig Research Center for Civilization Diseases (LIFE), 10,000 residents were included in this population-based cohort study. Detailed description of the cohort was described previously [23]. The main objective of the LIFE-Adult Study is to investigate prevalence, early onset markers, genetic predispositions as well as the role of lifestyle factors of major civilization diseases, especially metabolic and vascular diseases, heart function, cognitive impairment, depression, and allergies [23].

All data generated and analyzed during this study are included in this published article. The study was approved by the institutional ethics board of the Medical Faculty of the University of Leipzig. All methods were performed in accordance with the relevant guidelines and regulations. Written informed consent according to the Declaration of Helsinki was obtained from all individual participants included in the study. The authors had full access to all data in the study and take responsibility for its integrity and the data analysis.

All subjects underwent an extensive core assessment program (duration 5–6 h), including structured medical interviews and tests, physical examinations, and bio-specimen collections. Information about medication used by participants was collected.

**Fig 1:**
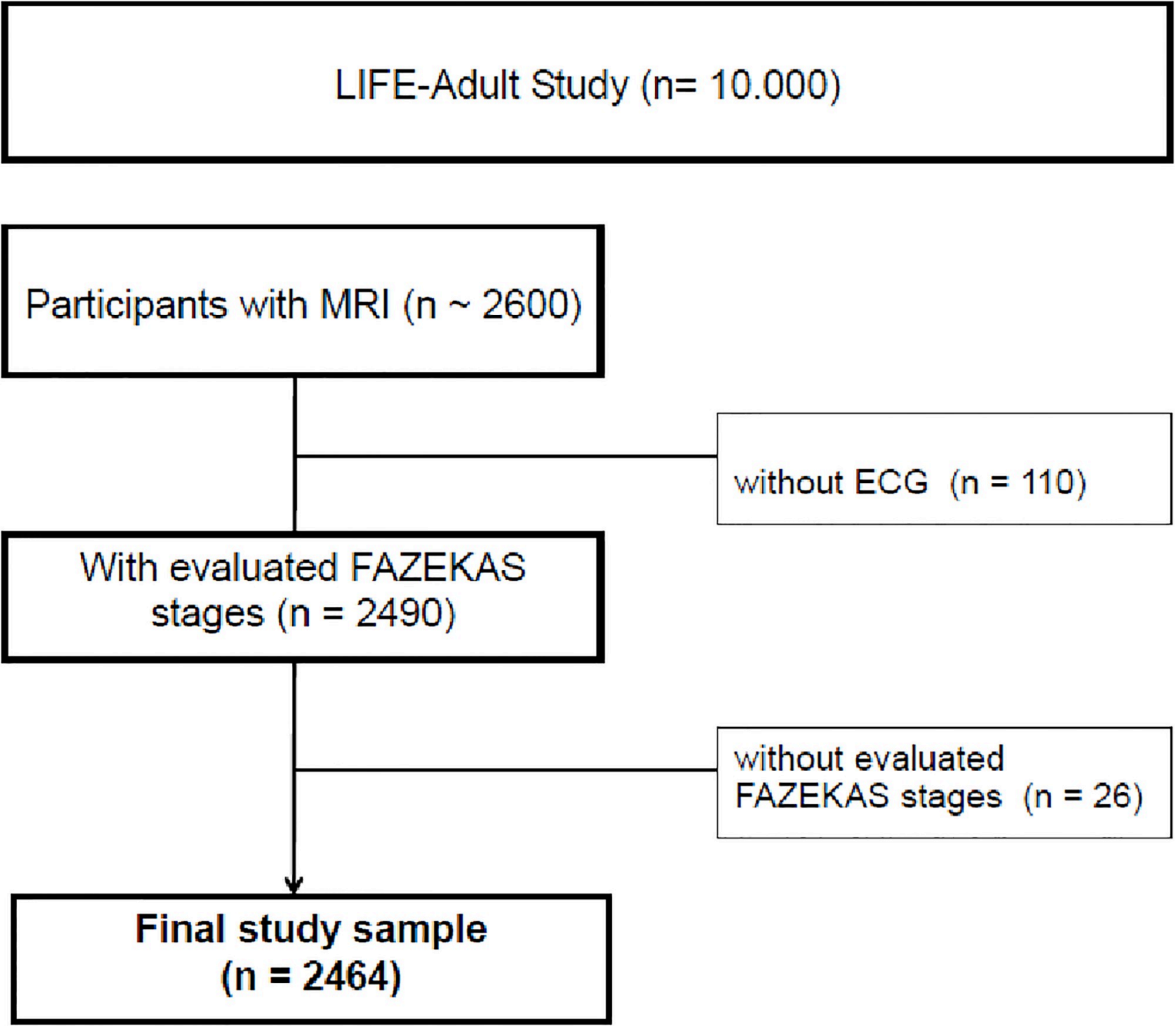
Flowchart of study population. 2636 participants underwent MRI imaging. 2464 participants had MRI imaging und ECG. The study sample of 2464 includes participants with evaluated FAZEKAS stages and ECG.

We included subjects who underwent ECG and MRI assessments. Antiarrhythmic drugs were defined as atrio-ventricular conduction decelerating medication (beta-blockers, dihydropyridine calcium channel blockers, class Ic and III antiarrhythmic drugs) [22]. MRI imaging was performed within 3 days in a subset of 2636 participants. Subjects with contraindication for MRI study (e.g., implanted pacemakers, joint implants), absent FLAIR image, and with bad image quality [7] were excluded from the study. A total of 2464 individuals were included in the analysis (Fig 1).

### Electrocardiographic analysis (ECG)

ECG analysis is described elsewhere [23]. Briefly, a 10-second 12-lead ECG was recorded using the PageWriter TC50 ECG system (Philips Medical Systems DMC GmbH, Hamburg, Germany) after a supine resting period of at least 10 min. The ECG of each participant was manually evaluated based on published criteria with particular focus on rhythm and conduction disturbances, ST-segment and J-point changes, T and U waves, PR and QT interval, hypertrophy, and QRS morphology [24]. AF was defined as irregular atrial rhythm with f-waves, documented in resting ECGs recorded at the research center. PR length was measured in milliseconds and categorized as PR prolongation if PR interval was measured >200 ms.

### Neuroimaging and assessment of white matter hyperintensities

WMHs were assessed on T2-weighted fluid attenuated inversion recovery (FLAIR) MRI scans with the four-stage Fazekas classification, which ranges from 0 to 3 [25]. All cMRI were performed with the same scanner (3-Tesla MAGNETOM Verio Scanner, Siemens, Germany). T1-weighted MPRAGE and FLAIR images were performed as part of a standardized protocol: MPRAGE [flip angle (FA) = 9°, relaxation time (TR) = 2300 ms, inversion time (TI) = 900 ms, echo time (TE) = 2.98 ms, 1 mm isotropic resolution, acquisition time (AT) = 5.10 min]; FLAIR (TR = 5,000 ms, TI = 1800 ms, TE = 395 ms, 1 × 0.49 × 0.49 mm resolution, AT = 7.02 min). Neuroradiologists were trained to rate WMHs using the Fazekas stages and were blinded to the individual’s diagnosis. WMHs can be divided into two subgroups depending on their location. A distinction was made between periventricular WMHs (PVWMHs), which run along the ventricular systems, and deep WMHs (DWMHs), which are located in the subcortical white matter [26].

Punctiform lesions in the brain parenchyma represent perivascular enlargements without substantial tissue damage. Confluent lesions, on the other hand, can show progression over time and lead to incomplete ischemic tissue destruction [25]. The Fazekas stage classification is the most frequently applied assessment tool for WMHs in everyday clinical care, enabling reliable quantification of WMHs. They assess the overall burden of cerebral WMHs ranging from zero (no WMHs) to three (severe WMHs) [27].

### Laboratory measurements

Blood was drawn from all study participants after fasting >8 hours and was analyzed on the same day. All samples were processed in a highly standardized manner; details are described elsewhere [23]. Laboratory measurements of creatinine, troponin T and NT-proBNP serum concentrations were performed on the same day at the Institute of Laboratory Medicine, University Hospital Leipzig (accredited by ISO 15189 and 17025) according to the Quality Standards for Medical Laboratories of the German Chamber of Physicians (RiLiBÄK) using assays from Roche Diagnostics on Cobas 6000 or 8000 (Roche Diagnostics) clinical chemistry analyzers.

### Statistical analysis

Baseline characteristics were described as medians and interquartile ranges (25^th^ and 75^th^ percentiles) for continuous variables as well as absolute and relative frequencies for categorical variables. Comparisons of continuous variables were made using non-parametric tests (Mann-Whitney-U-tests). Unordered categorical variables were compared using the Pearson χ2 test. Spearman correlation analysis was used to examine possible association between P-wave and PR interval.

Logistic regression analysis was used for uni- and multivariable analyses to identify factors associated with WMHs. Because PR interval is not measurable in patients with AF, we did not include this variable into analysis. In multivariable analyses, ECG parameters were adjusted for age, sex, and other clinically relevant parameters associated with WMHs. In addition to age and sex, *Model 1* included previous stroke, systolic and diastolic blood pressure, and PR interval. *Model 2* included all variables from Model 1 and the cardiac biomarker NT-proBNP, while *Model 3* included all variables from Model 1 and troponin T.

**Fig 2:**
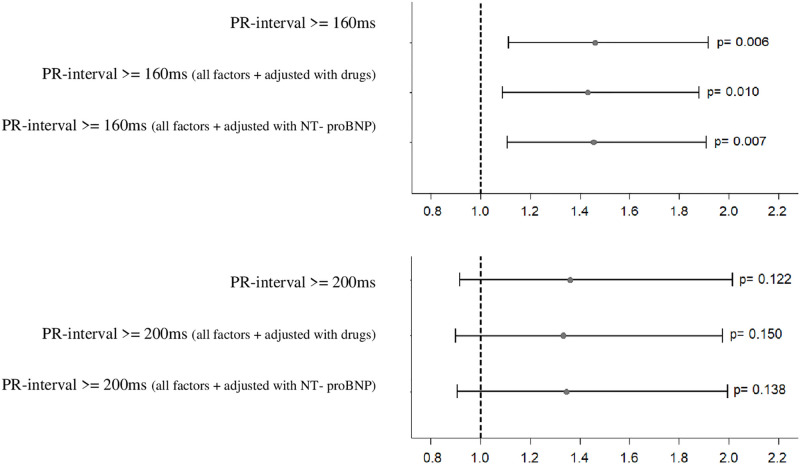
Multivariable analysis. Six multivariable models with 2 different cut-offs adjusted for clinically relevant factors for WMHs. Model 1: PR interval (cut-off 160 ms) adjusted for previous stroke, systolic and diastolic blood pressure; Model 2: Model 1 with further adjustment for cardiac medication; Model 3: Model 1 with further adjustment for NT-proBNP; Model 4: PR interval (cut-off 200 ms) adjusted for previous stroke, systolic and diastolic blood pressure; Model 5: Model 4 with further adjustment for cardiac medication; Model 6: Model 4 with further adjustment for NT-proBNP. PR interval ≥160 ms was associated with WMHs in all the multivariable models, whereas PR interval ≥200 ms was not. Odd’s ratio with 95% confidence interval and the respective P-value are shown.

Two cut-offs for the PR interval were chosen: first cut-off was 160 ms as the median value in our cohort, and second cut-off was 200 ms as the definition of PR interval prolongation. In Fig 2 we present six multivariable models with 2 different cut-offs as mentioned above adjusted for other clinically relevant factors for WMHs: (Model 1) PR interval (cut-off 160 ms) adjusted for previous stroke, systolic and diastolic blood pressure; (Model 2) Model 1 with further adjustment for cardiac medication; (Model 3) Model 1 with further adjustment for NT-proBNP; (Model 4) PR interval (cut-off 200 ms) adjusted for previous stroke, systolic and diastolic blood pressure; (Model 5) Model 4 with further adjustment for cardiac medication; (Model 6) Model 4 with further adjustment for NT-proBNP.

All statistical analyses were performed with SPSS Statistics 25. A p-value < 0.05 was considered statistically significant.

## Results

**Fig 3:**
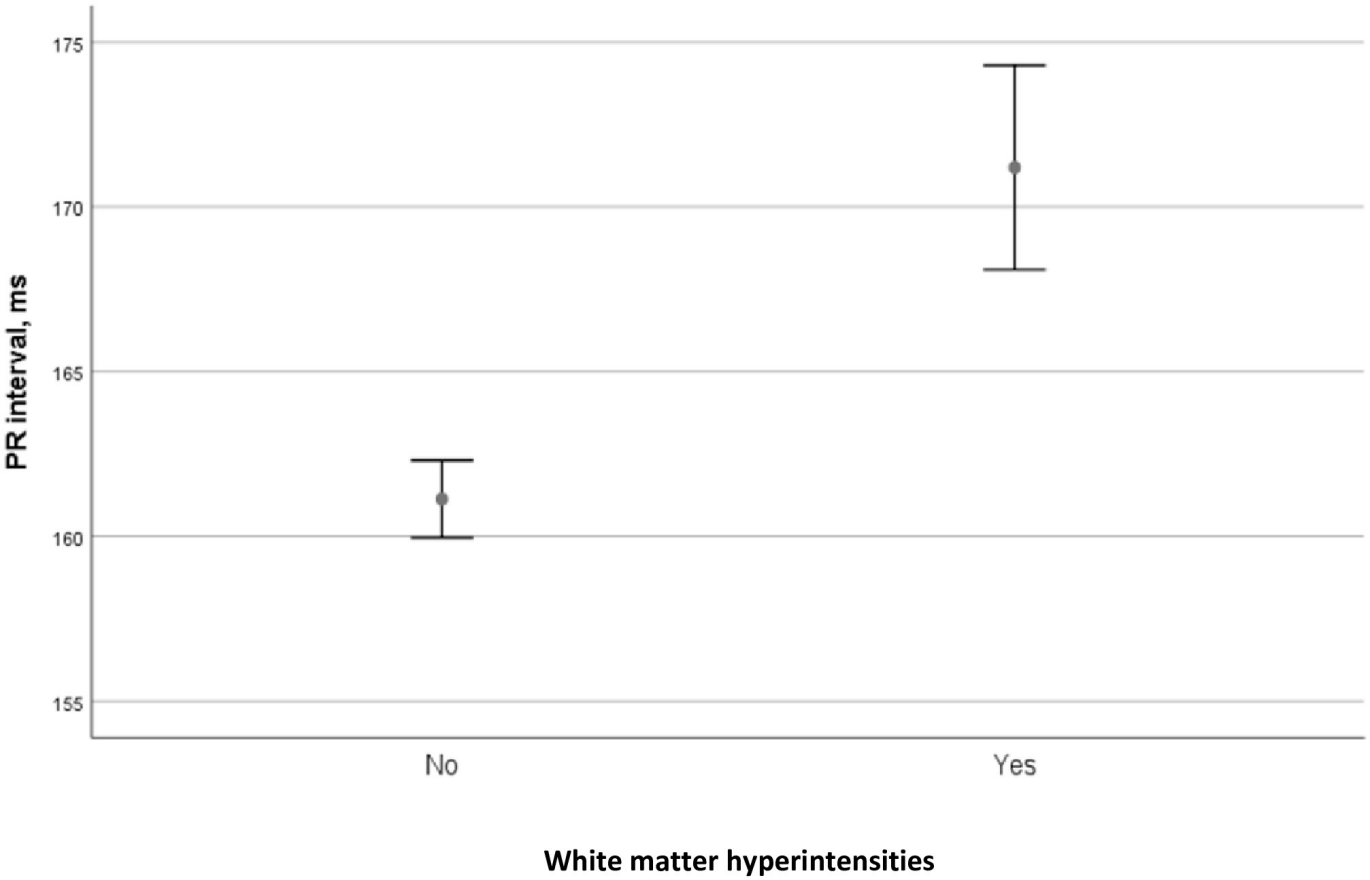
Association between PR interval duration and WMHs. The figure demonstrates association between white matter hyperintensities (*No*—Fazekas stage 0–1, *Yes*—advanced Fazekas stages 2–3) and PR interval length.

The study population comprised 2464 individuals. The baseline characteristics are presented in Table 1. The median PR interval was 160ms (interquartile range 143–179). There were 319 (13%) individuals with advanced WMHs. These individuals were significantly older and more often obese, with hypertension, diabetes, previous myocardial infarction, and stroke compared to patients without WMHs (all p <0.005). Patients with WMHs had higher levels of NT-proBNP, Troponin T, creatinine, cholesterol, and triglycerides (all p<0.05). Both PR interval and P-wave duration were higher in individuals with advanced WMHs compared to individuals without (Table 1, Fig 3). In the multivariable analysis, age (OR 1.10, 95% CI 1.08–1.12, p<0.001), systolic blood pressure (OR 1.01, 95% CI 1.01–1.03, p<0.004), and previous stroke (OR 2.86, 95% CI 1.5–5.47, p< 0.002) were associated with advanced WMHs (Table 3). Diabetes was not significant and not relevant (OR 1.03, 95% CI 0.73–1.47, p = 0.858) in multivariable analysis. Previous myocardial infarction was also not significant (OR 1.51, 95%CI 0.61–3.70, p = 0.370).

**Table 1:** Baseline characteristics of the study population.

	Total population	White matter lesions		p-value
Variables		None or not relevant (Fazekas 0–1)	Beginning or large confluence (Fazekas 2–3)	
	n = 2464	n = 2132	n = 332	
Age, years	64 (47; 70)	63 (46; 70)	71 (67; 75)	<0.001
Female	1144 (46.4%)	1001 (47.0%)	143 (43.1%)	0.185
Obesity (BMI≥30 kg/m^2^)	1627 (66.2%)	1377 (64.7%)	250 (75.5%)	<0.001
Systolic BP, mmHg	129 (119; 140)	128 (118; 139)	138 (127; 150)	<0.001
Diastolic BP, mmHg	75 (69; 81)	75 (68; 81)	77 (71; 83)	<0.001
Diabetes mellitus	269 (11.1%)	211 (10.0%)	58 (17.8%)	<0.001
Current smoking	361 (15.3%)	321 (15.6%)	40 (13.0%)	0.241
Previous stroke	51 (2.1%)	32 (1.5%)	19 (5.8%)	<0.001
Previous MI	26 (1.1%)	17 (0.8%)	9 (2.7%)	0.002
NT-pro BNP, pg/ml	68 (36; 124)	65 (34; 114)	106 (56; 182)	<0.001
Troponin T, pg/ml	5 (3; 7)	5 (3; 7)	7 (5; 10)	<0.001
Cholesterol mmol/l	5.6 (4.9; 6.3)	5.6 (4.9; 6.3)	5.8 (4.9; 6.4)	0.023
Triglyceride mmol/l	1.2 (0.8; 1.6)	1.2 (0.8; 1.6)	1.2 (0.9, 1.7)	0.018
Creatinine μmol/l	80 (70; 91)	80 (70; 91)	83 (71; 93)	0.013
P wave duration, ms	64 (58; 71)	64 (58; 71)	66 (60; 74)	<0.001
PR interval duration, ms	160 (143; 179)	159 (141; 177)	170 (152; 186)	<0.001
PR ≥200 ms	188 (7.9%)	145 (6.9%)	43 (13.5%)	<0.001

**Abbreviations**: BMI—body mass index; BP—blood pressure; MI—myocardial infarction.

Data presented as n (%) or median (interquartile range, 25^th^ and 75^th^ percentile)

### PR interval and white matter hyperintensities

In the univariable analysis, P-wave duration (OR per ms 1.01, 95% CI 1.01–1.02, p = 0.010) and PR interval (OR per ms 1.01, 95% CI 1.01–1.02, p<0.001) were significantly associated with advanced WMHs (Table 2). In the multivariable analysis, after adjustment for age, previous stroke, and systolic blood pressure, PR interval duration (OR 1.01, 95%CI 1.00–1.01, p = 0.034) was significantly associated with advanced WMHs (Table 3). However, the P-wave duration was not associated with advanced WMHs after adjustment for age and sex (OR 1.00, 95% CI 0.99–1.01, p = 0.607) and after further adjustment for other risk factors (OR 1.00, 95% CI 1.00–1.01, p = 0.476). We observed similar results in Model 2 for PR ≥200 ms after adjustment for NT-proBNP (OR 1.01, 95% CI 1.00–1.01, p = 0.043) and in Model 3 after further adjustment for troponin (OR 1.01; 95%CI 1.00, 1.01, p = 0.049) (Table 3). Using cut-offs for PR interval, the PR interval ≥160 ms was associated with WMHs in all the multivariable models, whereas PR interval ≥200 ms was not (Fig 2).

**Table 2:** Prediction of advanced WMHs (Fazekas stage 2–3), univariable (unadjusted) analysis.

Variables	OR	95% CI	p-value
Age, years	1.11	1.09, 1.13	<0.001
Women	0.86	0.68, 1.10	0.230
Previous stroke	3.82	2.08, 7.01	<0.001
Systolic BP, mmHg	1.03	1.03, 1.04	<0.001
Diastolic BP, mmHg	1.02	1.01, 1.03	0.004
NT-pro BNP, pg/ml	1.00	1.00, 1.00	<0.001
PR interval duration, ms	1.01	1.01, 1.02	<0.001
P wave duration, ms	1.01	1.00, 1.02	0.010
Heart failure	4.40	2.10, 9.22	<0.001

**Abbreviations**: as in Table 1; OR—odds ratio (presented per 1 unit—1 year, 1 mmHg, 1 pg/ml, 1 ms), CI—confidence interval

**Table 3:** Prediction of advanced WMLs (Fazekas stage 2–3), multivariable analysis.

Variables	Model 1			Model 2			Model 3		
	OR	95% CI	p-value	OR	95% CI	p-value	OR	95% CI	p-value
Age, years	1.10	1.08, 1.12	<0.001	1.10	1.08, 1.12	<0.001	1.10	1.08, 1.12	<0.001
Sex	1.14	0.87, 1.50	0.345	1.11	0.84, 1.47	0.459	1.21	0.92, 1.60	0.169
Previous stroke	2.86	1.49, 5.47	0.002	2.87	1.51, 5.55	0.001	3.01	1.60, 5.71	0.001
Systolic BP, mmHg	1.02	1.01, 1.03	0.004	1.01	1.00, 1.03	0.007	1.02	1.01, 1.03	0.004
Diastolic BD, mmHg	1.02	1.00, 1.04	0.062	1.02	1.00, 1.04	0.054	1.02	1.00, 1.04	0.040
PR interval duration, ms	1.01	1.00, 1.01	0.034	1.01	1.00, 1.01	0.043	1.01	1.00, 1.01	0.049
NT-pro BNP, pg/ml				1.00	1.00, 1.00	0.247			
Troponin T, ng/ml							1.02	1.00, 1.04	0.085

**Abbreviations**: as in Tables 1 and 2.

Model 1: after adjustment for age, previous stroke, and systolic blood pressure, PR interval duration is significantly associated with advanced WMHs. Model 2: after adjustment for NT-proBNP results stay similar. Model 3: after adjustment for troponin, results stay similar

### Heart failure, PR interval, and white matter hyperintensities

There were 30 participants (1.2%) with heart failure (HF). In the univariable analysis, the risk for advanced WMHs was 4.4-fold in participants with HF (Table 2). After adjustment for age and sex, stroke (OR 2.90, 95% CI 1.52–5.56, p = 0.001), hypertension (OR 2.06, 95% CI 1.57–2.70, p<0.001), HF (OR 2.88, 95% CI 1.27–6.55, p = 0.012), and PR interval ≥160ms (OR 1.40, 95% CI 1.05–1.86, p = 0.022) were associated with increased risk of advanced WMHs [28].

## Discussion

In this cross-sectional analysis, we demonstrate significant associations between PR interval duration and WMHs. We found that individuals with advanced stages of WMHs had longer PR interval duration than patients without WMHs. A significant association between WMHs and P-wave/PR interval prolongation was found in the univariable analysis.

In the multivariable analysis, only PR interval ≥160 ms demonstrated robust association with WMHs in different statistical models. Our study shows moderate correlation between P waves duration and the length of the PR interval (Spearman correlation coefficient = 0.5).

ECG is an easily available, cost effective, and informative diagnostic tool in clinical care. ECG quantifies the magnitude and direction of electric propagation and depolarization. The PR interval reflects comprehensive atrial conduction from sinus node activation through the atrioventricular node, while atrial and interatrial electrophysiological conductions generate the P-wave. Magnani et al. reported a significant association between P-wave duration and increased cardiovascular and all-cause mortality [19]. In addition to PR interval prolongation, the P-wave duration and characteristics are considered electrocardiographic endophenotypes for AF. However, the association between P-wave and its prolongation and WMHs is understudied. A population-based longitudinal analysis identified multiple P-wave indices that are associated with increased risk for adverse cardiovascular outcomes [29]. Increased atrial pressures due to structural heart disease also potentiate prolongation of P-wave indices. These disease states seem to share common pathways of atrial inflammation and fibrosis leading to atrial remodeling [30]. Atrial cardiomyopathy reflects abnormalities in left atrium including alterations in its structure (on macro- and micro-level) and function (contractility) [31]. Atrial activation could be measured by changes in electrocardiographic P-wave indices (PWIs), which could be considered as an electrophysiological component of atrial cardiomyopathy. P-wave axis, P-wave duration, advanced interatrial block, signal averaged P-wave, and P-wave terminal force in lead V1 (PTFV1) are some examples of PWIs [32]. It had been shown that PWIs are associated with incident AF [33], ischemic stroke [34, 35], and sudden cardiac death [36].

In our study, we found the association between P-wave and WMHs only in univariable analysis. Other studies with more advanced P-wave analyses (e.g., signal averaged ECG) could be helpful proving our results. The non-invasive signal averaged electrocardiogram (SAECG) uses a vector composite of filtered orthogonal leads and accurately measures cardiac activation times, including delayed atrial conduction [37, 38]. In comparison with the more common 12-lead ECG analysis, the SAECG could be superior in detection of P-wave prolongation as a risk factor of AF and related complications. However, this has not been analyzed to date and should be addressed in future studies.

Previously, we demonstrated that individuals in a large epidemiological cohort with PR interval prolongation and AF show similarities in echocardiographic parameters, renal function, and blood biomarker levels [23]. In the current analysis, we also considered PR interval prolongation as an intermediate phenotype for AF. Our results are in accordance with findings from the ARIC and the Framingham Heart Study, where an association between AF and WMHs was not found [4, 39].

WMHs have been hypothesized to be ischemic complications of Cerebral small vessel disease (SVD), based on histopathological studies that demonstrate small vessel changes in brains with WMHs [40]. These findings have been confirmed in clinical studies reporting associations between WMHs and microvascular risk factors such as hypertension [41] and diabetes [42], but not atherosclerosis [43]. Due to athersclerotic vascular changes, diabetes and prediabetes play an important role in the development of WMHs [44, 45]. However, in our study, we did not find the association between WMHs and diabetes.

Cerebral small vessel disease is one of the pathological mechanisms by which AF could cause cognitive impairment. White matter loss, hyperintensities, and impaired microstructural integrity are all MRI-based markers of small vessel disease. They are also predictors of cognitive function decline in older individuals [46]. Adverse effects of AF on cerebral small vessel disease may be one mechanism by which AF contributes to cognitive impairment. However, several studies failed to demonstrate an association between AF and WMHs [28, 47, 48]. Of note, a cross-sectional analysis of the community-based Framingham Heart Study did not report any associations between AF and lower total brain volume [7]. Incident AF is associated with structural changes in brain MRI, including worsened sulcal grade, larger ventricles, and subclinical cerebral infarctions. However, analyses from the ARIC and the Framingham Heart Study could not demonstrate any association between AF and WMHs or total brain volume after adjustment for vascular risk factors [39, 47, 49]. Notably, WMHs, attributed to small vessel disease and global cerebral hypoperfusion, were not associated with AF [49]. Our results confirm previous findings. Compared to individuals with normal PR interval, the association between WMHs and individuals with AF was not significant.

In addition to microangiopathy, cerebral hypoperfusion caused by left ventricular dysfunction may contribute equally to the development of structural and functional cerebral abnormalities in HF patients [10].

## Strengths and limitations

The strength of the study is sample size. Of the 10,000 individuals recruited to the LIFE-Adult-Study, over 2600 had received a brain MRI; of these, 2464 individuals also had an ECG.

Values for the majority of the subgroup came out normal because patients impaired by illness and with motor disability were more likely to refrain from participation.

Because of the cross-sectional nature of our analysis, the finding of PR interval duration associated with WMHs allows only for hypothesis generation; no causal relationship could be determined. Further longitudinal studies are needed to analyze whether both factors could modulate the occurrence of dementia and AF. This study is a single center observational study of individuals with European ancestry from a small geographic area in Eastern Germany. Thus, generalizability of the study results to individuals of other races/ethnicities is limited. Finally, our findings should be confirmed by other studies with continuous ECG monitoring, advanced P-wave indices, and PR interval analyses.

## Conclusions

We found that the length of PR interval is associated with advanced WMHs. Overall, a complex interplay of factors lead to cerebral injury through various mechanisms affecting the cerebral macro- and microenvironments. However, the results indicate that ECG—a simple diagnostic tool—could be used identifying individuals at higher risk for WMHs. Nevertheless, despite mostly hypothesis generating findings, further studies are needed to prove our results in longitudinal setting.
